# Biomechanics and mechanobiology of the bone matrix

**DOI:** 10.1038/s41413-022-00223-y

**Published:** 2022-08-30

**Authors:** Chunyang Ma, Tianming Du, Xufeng Niu, Yubo Fan

**Affiliations:** 1 Key Laboratory of Biomechanics and Mechanobiology (Beihang University), Ministry of Education, Beijing Advanced Innovation Center for Biomedical Engineering, School of Biological Science and Medical Engineering, Beihang University, Beijing, 100083 China; 2 Department of Biomedical Engineering, Faculty of Environment and Life, Beijing University of Technology, Beijing, 100124 China; 3 Research Institute of Beihang University in Shenzhen, Shenzhen, 518057 China; 4 School of Engineering Medicine, Beihang University, Beijing, 100083 China

**Keywords:** Bone quality and biomechanics, Bone

## Abstract

The bone matrix plays an indispensable role in the human body, and its unique biomechanical and mechanobiological properties have received much attention. The bone matrix has unique mechanical anisotropy and exhibits both strong toughness and high strength. These mechanical properties are closely associated with human life activities and correspond to the function of bone in the human body. None of the mechanical properties exhibited by the bone matrix is independent of its composition and structure. Studies on the biomechanics of the bone matrix can provide a reference for the preparation of more applicable bone substitute implants, bone biomimetic materials and scaffolds for bone tissue repair in humans, as well as for biomimetic applications in other fields. In providing mechanical support to the human body, bone is constantly exposed to mechanical stimuli. Through the study of the mechanobiology of the bone matrix, the response mechanism of the bone matrix to its surrounding mechanical environment can be elucidated and used for the health maintenance of bone tissue and defect regeneration. This paper summarizes the biomechanical properties of the bone matrix and their biological significance, discusses the compositional and structural basis by which the bone matrix is capable of exhibiting these mechanical properties, and studies the effects of mechanical stimuli, especially fluid shear stress, on the components of the bone matrix, cells and their interactions. The problems that occur with regard to the biomechanics and mechanobiology of the bone matrix and the corresponding challenges that may need to be faced in the future are also described.

## Introduction

Bone is an important organ that provides mechanical support for the human body, and the bone matrix is an important component of bone^1–3^. For a long time, the bone matrix has attracted extensive attention because of its superior role in the human body and unique mechanical properties^4–7^. As a natural biomaterial, the bone matrix has superior mechanical properties compared with synthetic materials. Studies on the biomechanical properties and biological significance of the bone matrix, as well as the composition and structure of the bone matrix, will shed light on bone substitute implants, biomimetic bone materials, scaffolds for human bone tissue repair, and biomimetic applications in other fields^8–10^. Moreover, the bone matrix can be affected by many factors in its surrounding environment, especially mechanical stimulation, during both growth and health maintenance^11–13^. Through the study of the mechanobiology of the bone matrix, the response mechanism of the bone matrix to its surrounding mechanical environment can be elucidated and used to promote the health maintenance of bone tissue and defect repair.

It is difficult for synthetic materials to achieve both strength and toughness; that is, those with high strength usually show poor toughness, and those with strong toughness usually show low strength^4,14^. The bone matrix is mainly composed of hydroxyapatite (HA) and collagen (Col). HA accounts for ~40%–45% of the volume of the bone matrix, and Col accounts for ~55%–60%^14^. Among them, HA has high strength, and Col fibers have strong toughness^14^. As a natural biomaterial, the bone matrix achieves both strength and toughness because of its complex multilevel structure from the nano- to macroscale^4^. This complex multiscale structure and the biomechanical properties of the bone matrix provide a model for the synthesis of new composite biomaterials. Therefore, to study the biomechanics of the bone matrix while focusing on its excellent biomechanical properties, attention should be given to the biological significance of these mechanical properties, as well as the composition and structure of the bone matrix. Mastering each mechanical property of the bone matrix and its biological significance is a prerequisite for preparing more suitable bone substitute implants and scaffolds for human bone tissue engineering. Understanding the influences of the composition and structure of the bone matrix on its mechanical properties can provide a reference for the preparation of bionic materials and bone bionics in other fields.

The growth, repair, and health maintenance of human bones are affected by many factors in the surrounding environment. Mechanical stimulation is one of the most important environmental factors^11,13,15^. Human life activities, such as running, jumping, swimming, and bungee jumping, will lead to drastic changes in the mechanical environment around the bone matrix. Mechanical stimuli will have significant impacts on the components of bone matrix, Col and HA, as well as the cells living in the bone matrix, and then affect the interactions between them^11,13,15^. Common mechanical stimuli mainly include compressive stress, tensile stress and fluid shear stress (FSS)^15^. Among them, FSS has significant effects on the components of the bone matrix and cells^11,13,16^. In vivo, fluid shear stress mainly acts on the surfaces of the lacunar-canalicular walls and cell membranes, as well as the endosteal surface. In addition, the Col in newly formed osteoid and that lining the bone surface could also be exposed to fluid flow. According to the research of Weinbaum and Cowin et al., the space between the canalicular wall and the osteocyte process can lead to shear stresses of 0.5–3.0 Pa for mechanical loads in the physiological range^17^. In in vitro systems of nonbone tissue, FSS is able to promote the directional alignment of Col fibrils and directional growth of HA on the surface of Col fibrils. Cells growing on directionally aligned Col fibrils will grow directionally and secrete oriented extracellular matrix^16^. Studying the effects of mechanical stimuli, especially FSS, on Col, HA, cells, and the interactions among them may shed light on the defect repair and health maintenance of bone tissue.

**Fig. 1:**
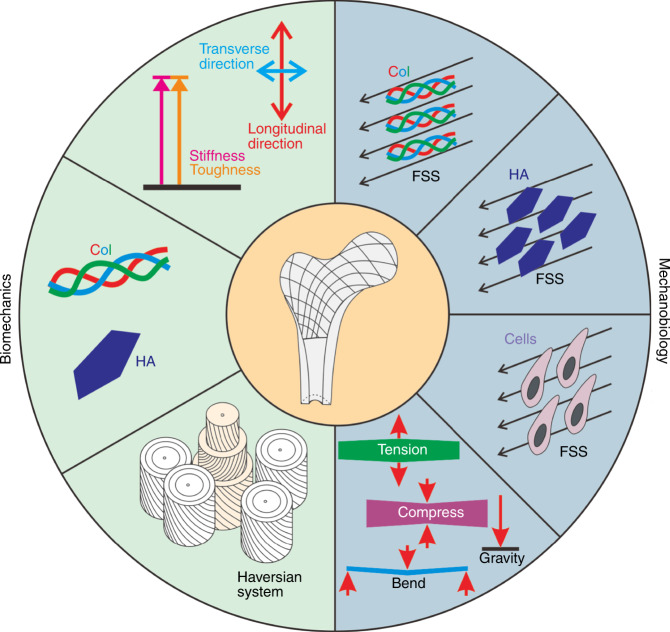
Schematic representation of the biomechanical properties of the bone matrix and the effects of mechanical stimuli on the bone matrix components, Col and HA, and bone-related cells, such as mesenchymal stem cells, osteocytes, osteoblasts, osteoclasts

This paper summarizes the biomechanical properties of the bone matrix and their biological significance, discusses these mechanical properties from both compositional and structural aspects, and studies the effects of mechanical stimulation, especially FSS, on bone matrix components, cells and their interactions (Fig. 1). The problems that occur with regard to the biomechanics and mechanobiology of the bone matrix and the challenges that may need to be faced in the future are described.

## Biomechanics of the bone matrix

Bone is an indispensable load-bearing tissue for the human body, and the bone matrix is a major component of bone. Over the years, bone matrix has gained much attention because of its excellent mechanical characteristics and complex multistage structure. This section summarizes the biomechanical properties of the bone matrix and their biological significance and explores how the composition and structure affect the mechanical properties of the bone matrix.

### Biomechanical properties of the bone matrix and their biological significance

**Fig. 2:**
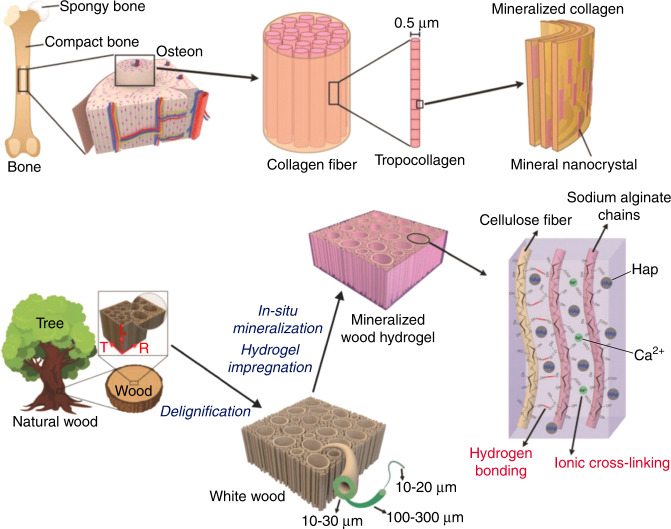
Biomimetic strategy for preparing highly anisotropic, ultrastrong and stiff osteoconductive mineralized wood hydrogel composites. Figures adapted with permission from ref. ^19^

Obvious mechanical anisotropy and high strength and strong toughness are two mechanical properties of the bone matrix, which have attracted extensive attention^4,6,14^. These characteristics are the result of a long period of natural evolution and play crucial roles in current human life activities. Elucidating the biomechanical properties of the bone matrix will help to deepen the understanding of the human bone matrix and provide a reference for the design of bone substitute implants and scaffolds for bone tissue engineering. The Young’s modulus in the longitudinal direction of cortical bone is ~15–25 GPa, which is significantly higher than its transverse Young’s modulus^18^. This mechanical property of the bone matrix has attracted wide attention and has been applied in the design of scaffolds for bone tissue engineering. For example, Wang^19^ impregnated sodium alginate hydrogel into delignified wood and then immersed it in 300 mmol·L^−1^ K_2_HPO_4_ solution, 500 mmol·L^−1^ CaCl_2_ solution, NH_3_·H_2_O solution with a pH value of 11 and phosphate-buffered saline sequentially to sequentially mineralize HAP in situ to prepare mineralized wood hydrogel composites with obvious mechanical anisotropy (Fig. 2). It is difficult for synthetic materials to attain both strength and toughness^4,14^. However, as a common natural biomaterial, the bone matrix is capable of high strength (10–20 GPa) and strong toughness (2–7 kJ·m^−2^) simultaneously^14^. This mechanical characteristic of the bone matrix provides a perfect reference for the development of artificial materials.

At the macrostructural level, bones in different parts of the human body, different positions of the same bone and different directions at the same position show different mechanical properties. Rho et al.^20^ and Marle et al.^21^ used ultrasonic technology to study the elastic modulus, shear modulus, Poisson’s ratio and density of some human bones (including femur, tibia, humerus, mandible, lumbar spine, and patella). The experimental results showed that there was no difference in the mechanical properties of the humerus, proximal tibia and lumbar spine. The stiffness and strength of cancellous bone among these types of bones were lower than those of the patella, distal femur and proximal femur (the cancellous bone of the patella has the highest value in general). Bonfield and Grynpas^22^ measured the Young’s modulus of bovine femur in different directions under dry and wet conditions. Turner CH^23^ used acoustic microscopy and nanoindentation to measure and compare the Young’s moduli of trabecular and cortical bone tissues from a common human donor. The experimental results showed that the Young’s modulus of cortical bone in the longitudinal direction was ~40% greater than the Young’s modulus in the transverse direction. The Young’s modulus of trabecular bone tissue was slightly higher than the transverse Young’s modulus of cortical bone but substantially lower than the longitudinal Young’s modulus of cortical bone. These findings were consistent for both measurement methods and suggest that the elasticity of trabecular tissue is within the range of that of cortical bone tissue. Rho et al.^20^ used ultrasonic technology and a microtensile test to determine the Young’s modulus of a single trabecular bone, and microsamples of cortical bone were cut into similar sizes to those of a single trabecular bone. The experimental results showed that the average trabecular Young’s modulus measured ultrasonically and mechanically was 14.8 GPa and 10.4 GPa, and the average Young’s modulus of the cortical bone microspecimens measured ultrasonically and mechanically was 20.7 GPa and 18.6 GPa. With either testing technique, the mean trabecular Young’s modulus was found to be significantly less than that of cortical bone. Barak et al.^24^ examined the question of whether the stiffness (Young’s modulus) of secondary osteonal cortical bone is different under compression and tension. The test results showed that the tensile Young’s modulus was slightly but significantly greater than the compressive Young’s modulus. Spatz et al.^25^ measured the Young’s modulus and shear modulus of cortical bone from mammalian, avian, and staghorn bone using three-point bending. The experimental results showed that the main determinant for the mechanical properties was the mineral content. For mammalian bone, the frequency of Haversian systems correlates negatively with stiffness and resistance to shear.

It has been shown that the mechanical properties of bone vary at different structural levels. Rho et al.^26^ described this in detail. For example, Reilly et al.^27^ measured and compared the elastic moduli of human bone and bovine bone specimens by compression and tensile tests. The experimental results showed that there was no significant difference between the moduli determined under the two loading modes. The Young’s modulus of large tensile cortical specimens has been shown to be in the 14–20 GPa range. Choi et al.^28^ measured the elastic moduli of the subchondral, trabecular and cortical bone tissue of human proximal tibia by a three-point bending test at the microstructural level. Eight cortical samples of different sizes (*h* = 100–1 000 μm) were used to examine the size dependence of the modulus. The experimental results showed that the average modulus of subchondral bone specimens was 1.15 GPa, and those of the trabecular and cortical specimens were 4.59 GPa and 5.44 GPa, respectively. There are significant differences between the modulus values of bone tissues, which may be mainly due to the different microstructures of each bone tissue rather than the different mineral densities. In addition, there is a significant correlation between modulus and sample size. Although the modulus value of relatively large samples remains quite constant (~15 GPa), the modulus value decreases as the sample size decreases. Rho et al.^29^ used the nanoindentation method to study the inherent elastic properties of several microstructural components of human vertebral trabecular bone and tibial cortical bone. The experimental results showed that the measurements of the vertebral trabeculae were made in the transverse direction, and the average Young’s modulus was found to be 13.5 ± 2.0 GPa. The tibial specimens were tested in the longitudinal direction, yielding moduli of 22.5 ± 1.3 GPa for the osteons and 25.8 ± 0.7 GPa for the interstitial lamellae. In addition, Ji and Gao’s experimental results showed that the elastic properties of bone can be highly anisotropic at the nanoscale^30^. The basic building blocks (HA crystals and Col fibrils) are extremely small, making mechanical testing nearly impossible. Therefore, according to the different levels or structures of bone materials, it can be supposed that the decomposition of bone mechanical tests deserves attention.

Organisms in nature have all evolved through a long period of natural evolution, and under the law of “survival of the fittest”, the various organs of organisms evolved in a way that allowed adaptation to their living environments. Bone is an important organ that provides mechanical support for the human body. In the process of human evolution, bone has also undergone significant changes. At present, the mechanical properties of human bone are closely related to the modes of human activities^31^. The Young’s modulus of cortical bone is significantly higher than that of cancellous bone and has obvious mechanical anisotropy^6,20,21^. The main reason for this is that the main roles of cortical bone and cancellous bone in the human body are different. Cortical bone is mainly involved in providing mechanical support. The Young’s modulus of cortical bone in the longitudinal direction is significantly higher than that in the transverse direction, which indicates that this bone resists more forces in the longitudinal direction during human activities. The bone matrix has both high strength and strong toughness, indicating that bone requires both high strength to deal with the impact of stress and strong toughness to resist the fracture and damage caused by deformation, which corresponds to the role of bone in the human body. Proteins account for ~50% of the volume of animal endoskeletons but only 5% of that of nacre, and the Young’s modulus of bone (10–20 GPa) is significantly less than that of shell (50 GPa)^14^. Ji and Gao^14^ pointed out that this difference might be because bone experiences more dynamic loads, such as compressive stress, and larger deformation during the lifetime of animals. Moreover, the shear modulus of cortical bone is only 5% of the Young’s modulus, and Spatz et al.^25^ argued that a lower shear modulus was not only a prerequisite for the control of crack propagation but also allowed hollow bones to react smoothly to local impacts, which otherwise may lead to failure.

The bone matrix has the mechanical properties described above, which results in high requirements for artificial bone substitute implants and scaffolds for bone tissue engineering. For example, implants with low Young’s moduli cannot provide sufficient mechanical support, but if their Young’s moduli are too high, the bone near the implant will be resorbed, so the mechanical properties of the implant should be as close as possible to those of the human bone matrix^32^. In addition to having mechanical properties similar to that of the bone matrix, the implant should also have a certain mechanical anisotropy. Wang et al.^18^ pointed out that the structure and properties related to anisotropy also played important roles in damage tolerance, cell guidance and differentiation, and the transmission of biological factors and nutrients. However, whether bone tissue engineering scaffolds with similar mechanical properties to those of the human bone matrix are the best choice for promoting bone regeneration is still uncertain. In addition, bone tissue engineering scaffolds must often have pores of different sizes to provide access and attachment surfaces for cells. How to simultaneously endow scaffolds with a certain number of pores and suitable mechanical properties remains to be solved.

### Influences of composition and structure on the biomechanics of the bone matrix

The excellent biomechanical properties of the bone matrix are determined by its composition and structure. Col has strong toughness and low strength, while HA has high strength and poor toughness. The complex multilevel structure of the bone matrix combines these two components to yield biomechanical characteristics of both toughness and strength. This section will analyze the biomechanical properties of the bone matrix from two aspects: composition and structure.

#### Effect of composition

**Fig. 3:**
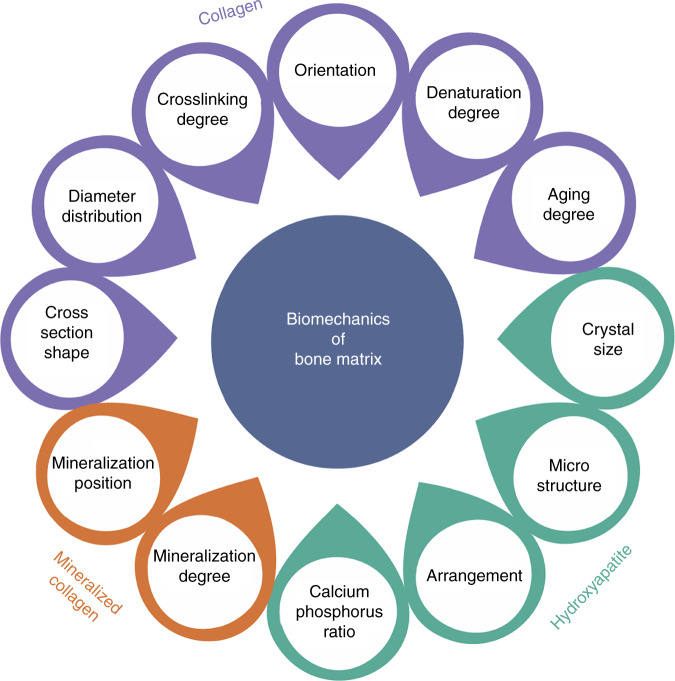
Component factors affecting bone-matrix biomechanics

HA and Col are the main components of the bone matrix. Without these two components, the bone matrix would not have such excellent mechanical properties. The mechanical properties of the bone matrix and its main components are shown in Table 1. The biomechanical properties of the bone matrix change with the diameter distribution, crosslinking degree, orientation, denaturation degree of Col fibers, as well as changes in other properties caused by aging. The mechanical strength of bone can be changed by controlling the crystal size, microstructure and calcium–phosphorus ratio of HA, while the mineralization degree of mineralized Col and the mineralization position of HA can also affect the biomechanical properties of the bone matrix. This section will analyze the effects of these components on the biomechanics of the bone matrix from 3 aspects: Col, HA and mineralized Col (Fig. 3).

**Table 1:** Mechanical properties of bone and its main structural components: Col fiber and calcium phosphate

	Stiffness/GPa	Toughness (kJ·m^−2^)
Col fiber	0.1–1	>10
Calcium phosphate	100	<0.1
Bone	10–20	2–7

**Fig. 4:**
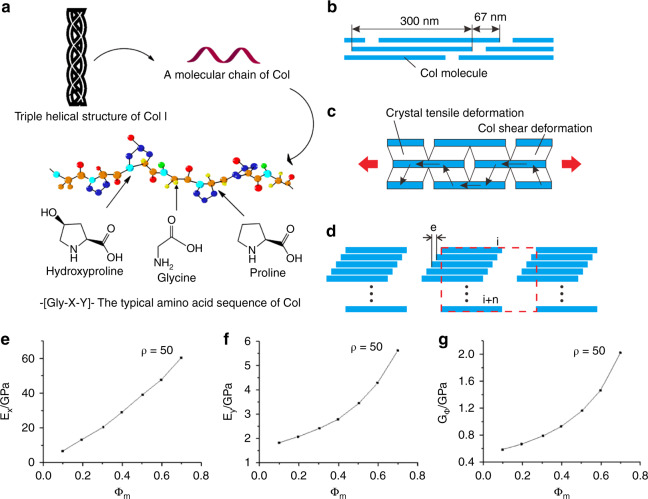
Structure and mechanical properties of Col fibers. **a** Special triple helix structure and amino acid sequence of type I Col^165^. **b** Mineralized Col fiber structure model. **c** Mineralized Col fiber stress transfer model. **d** Schematic of RVE that is indicated by the red dashed line. **e** Changes in longitudinal elastic modulus with degrees of mineralization changes. **f** Changes in lateral elastic modulus with degrees of mineralization changes. **g** Changes in shear elastic modulus with degrees of mineralization changes^166^. Figures adapted with permission from refs. ^165,166^

Collagenous proteins are a major constituent of all extracellular matrices^33^. The Col family has at least 28 members, and Ricard-Blum described it in detail^34^. The type of Col in the bone matrix is type I, accounting for ~55%–60% by volume and existing in the form of fibers. Type I Col has a special triple helix structure, which consists of two α1 chains and one α2 chain. Col fibers have the mechanical characteristics of strong toughness and low strength. The triple helix structure is the key to the strong toughness of Col^35^. The special triple helix structure and amino acid sequence of type I Col are shown in Fig. 4a.

The diameter distribution, cross-linking degree, orientation, denaturation degree of Col fibers and other changes that occur with aging have significant impacts on the biomechanical properties of the bone matrix^36,37^. The diameters of Col fibers or fibrils are different in different tissues and organs of the human body. For example, the diameter of Col fibers in normal tendon tissue is ~120 nm^38^, which is twice the diameter of Col fibers in many other tissues^39^. The diameter of Col fibers in the extracellular matrix is ~20 to hundreds of nanometers, and the diameter of fiber bundles can reach hundreds of micrometers^40^. The tensile strength of tissue containing type I Col is related to the diameters of the fibers and fibrils^41,42^. Christiansen DL’s experiment showed that when the strain of Col fiber was less than 20%, the diameter of Col fiber was positively correlated with its elastic modulus. When the strain of Col fiber was greater than 20%, there was no correlation between its diameter and elastic modulus. These results showed that the transverse aggregation of Col fibrils affects the mechanical properties of Col fibers when the strain of Col fiber was less than 20%^43^. In addition, local tissue lesions, such as canceration, will significantly change the diameter and cross-sectional shape of Col fibers, resulting in changes in the mechanical properties and other physical and chemical properties of diseased tissues. Therefore, Col abnormalities may be used to predict the emergence of diseases. At present, some relevant reports have been published^39,44^. However, to predict disease emergence through the observation of Col morphology, more in-depth and detailed research is needed. The crosslinking of Col also has a certain impact on the mechanical properties of Col and tissues with Col as the main component^45^. Thompson and Czernuszka^46^ compared the effects of two crosslinking methods on the mechanical properties of Col: (a) glutaraldehyde and (b) a combination of dehydrothermal treatment and cyanamide. The experimental results showed that crosslinking can increase the elastic modulus of wet Col from ~25–30 MPa to 55-60 MPa but has little effect on its fracture stress, and the strain to failure is reduced. Crosslinking reduced the work of fracture of Col. Crosslinking had the same effect on the elastic modulus, fracture stress, and strain to failure of dry Col but had no effect on the work of fracture. Paschalis EP’s experimental results also proved that Col crosslinks play a pivotal role in the determination of bone quality and mechanical integrity^47^. Martin and Ishida^48^ determined the effects of Col fiber orientation, porosity, dry/wet density and mineralization on the tensile strength of bovine cortical bone. The results showed that the orientation of Col fibers had a significant effect on the tensile strength of bovine cortical bone and can be used as a reliable index for predicting the tensile strength of the bone matrix. The density of the bone matrix, porosity and mineralization cannot be used to predict the tensile strength of the bone matrix. Wang et al.^49^ used a heating model to study the effect of Col denaturation on the biomechanical properties of human cadaveric bone. Bone specimens were heat-treated at different temperatures (37–200 °C) to induce different degrees of Col degeneration. The degree of Col degeneration and the mechanical properties of bone were measured by the selective digestion technique and three-point bending tests. The results showed that with increasing Col denaturation, the toughness and strength of bone decreased significantly, while the elastic modulus of bone was almost constant, which was unrelated to Col denaturation. These results showed that the Col network plays an important role in the toughness of bone but has little effect on the stiffness of bone. With the aging of the human body, the mechanical properties of the bone matrix deteriorate. Zioupos et al.^50^ studied the mechanical properties (stiffness, strength, and toughness) of human femoral bone and the changes in the Col properties (the concentration of stable mature crosslinks, the shrinkage temperature, and the rate of contraction during isometric heating) of the bone matrix at different ages (35–92 years old). The results showed that there was no significant correlation between the changes in Col in terms of the concentration of mature (pyridinium and deoxypyridinium) crosslinks and the changes in age and the mechanical properties of the bone matrix. The shrinkage temperature decreased with age and was related to the toughness of the bone matrix. The maximum rate of contraction was strongly correlated with the three different measures of tissue toughness but much less correlated with stiffness and strength. The results reinforced speculation regarding the toughening role of Col in bone mechanics and suggested that the fragility of aging bone may be related to Col changes. Banse X’s experimental results also proved that the properties of the organic matrix in adult vertebral cancellous bone can be changed, and these changes affect the mechanical properties of the bone matrix^51^.

HA accounts for 40%–45% of the volume of the bone matrix and is a key component for enhancing the mechanical properties of the bone matrix. The crystal structure of HA can indicate trabecular bone quality by the identification of crystallite size, microhardness, microstrain, and ratio of calcium and phosphorus in three types of bone: normal, osteopenic, or osteoporotic^52^. Through the exploration of the bone microstructure and HA crystal structure, Rollo et al.^52^ found that bone density and mechanical strength can be reflected by the size, microstrain and calcium–phosphorus ratio of HA crystals. The results showed that the crystallite size, hardness, calcium–phosphorus ratio, trabecular number and bone mineral density decreased, and the microstrain value increased, indicating that the crystal structure of HA in osteoporotic and osteoporotic bone was more fragile and damaged^52^. Moreover, the HA coating also affects the change and distribution of implant-bone interface stress. Jiang et al.^53^ studied the effect of HA coating on the stress distribution at the implant-bone interface and found that the stress distribution near the interface decreased with increasing HA coating thickness, and the biomechanical properties of bone improved. However, when the HA coating thickness was 60–120 μm, the difference in stress reduction was no longer obvious. The shear strength between the bone and plasma-sprayed HA coating is affected by the plasma spray thickness. Yang and Yang^54^ sprayed Ti_6_Al_4_V columns with HA coatings (HACs) with thicknesses of 50 μm and 200 µm and implanted them into canine femurs. The push-out testing of the implant bone interfaces proved that when the HAC thickness was 50 μm, the shear strength was higher. The plasma-sprayed HACs exhibited compressive residual stresses, and the thicker HACs exhibited higher residual stresses than the thinner HACs. Based on these results, some studies have been conducted to try to study the effects of the size and structure of a single HA crystal on the resulting mechanical properties. Libonati et al.^55^ studied the effect of geometric confinement on the fracture mechanism of the HA crystals that form the mineralized phase in bone. The experimental results showed that when the height of the bone-mineralized HA crystals was less than 4.15 nm, the stress concentration at the tip of the crack disappeared, showing a strong toughness and large stress-carrying capacity. In addition, HA nanoparticles can improve the crystallinity, shear resistance and thermodynamic properties of a polypropylene lactone/chitosan mixture^56^. Therefore, the crystal structure and mechanical properties of HA will be popular research topics in the future.

In the process of mineralization, calcium and phosphorus ions accumulate in Col fibers, convert into HA, and finally form mineralized Col fibers^57^. The mineralization degree of Col fiber has an important impact on the mechanical properties of Col fiber (Table 2)^58,59^. The stress transfer, longitudinal elastic modulus, transverse elastic modulus, and shear elastic modulus change with the mineralization degree of Col (as shown in Fig. 4b, c). Mineralized Col fibril can be represented by a representative volume element (RVE) composed of *n* subunits, as shown in Fig. 4d. The elastic modulus of mineralized Col fibers decreases with an increasing number of the subunits of fiber RVEs. However, when the mineral volume fraction is low, RVEs with more subunits can obtain higher elastic moduli by adjusting the distribution of the Col matrix^60^. The proline-related Raman band showed an obvious stress response of the mineralized Col, which was consistent with the progressive linkage of the Col triple helix and HA nanocrystal mineralization^61^. The concentration of calcium and the phosphorylation degree of Col also affect the mineralization of Col and then affect the mechanical properties of the bone matrix. Niu et al.’s study showed that a low concentration of calcium was beneficial to Col assembly but inhibited mineral crystallization, and a high concentration of calcium hindered Col self-assembly, whereas it benefited mineral crystallization^62^. Du et al.’s study showed that the chelating amount of calcium was improved linearly with an increasing phosphorylation degree of Col fibrils, which demonstrated that the introduced phosphate groups served as new nucleation sites and participated in the formation of apatite minerals inside the Col fibrils^63^. In addition, Dinesh R. Katti and Kalpana S. Katti used molecular dynamics (MD), steered MD and steered MD simulations to study the load-carrying behavior of Col in the proximity of HA^64,65^ and the directional dependence of the deformation response of Col with respect to the HA surface^66^. These research results showed that the interface between HA and Col affected the overall load–deformation response of Col; water significantly influenced the load-deformation response of Col due to Col-water HA interactions; the mineral influenced interactions between solvated tropocollagen in a HA-tropocollagen system; and the mechanics of Col pulled in different directions with respect to HA were significantly different. The mechanical properties of Col fibers with different mineralization positions, such as Col mineralized outside the fiber and Col mineralized inside the fiber, also need to be further studied in in vitro biomineralization.

**Table 2:** Effects of mineralization degree on the mechanical properties of Col

Col type	Degree of mineralization	Young’s modulus/MPa	Maximum elastic strain/%	Maximum elastic stress/MPa
Pure Col	0	50	35	20
Tendon Col	0.15	400–700	6–8	30–40
Bone Col	0.40–0.45	10 000–20 000	0.5–1.0	100

#### Effect of structure

**Fig. 5:**
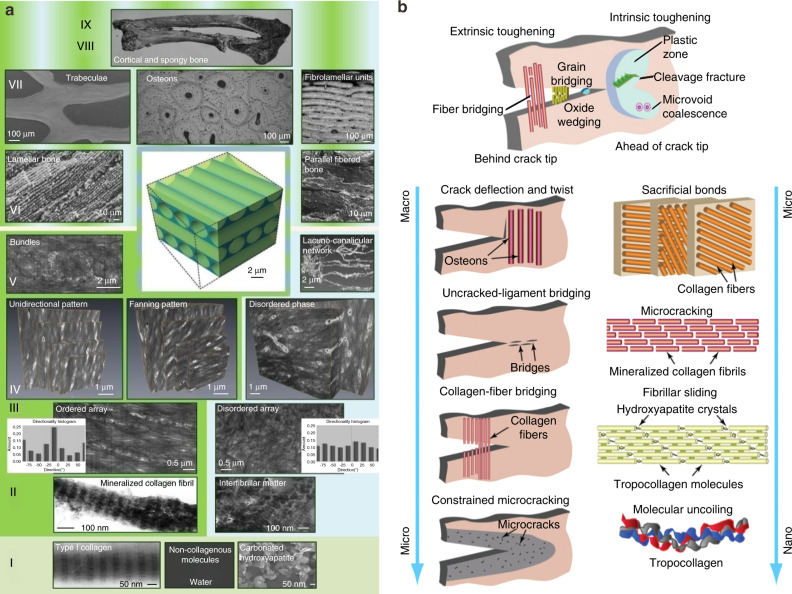
Multistage structure of bone matrix and its toughening mechanism. **a** Nine-level structure of bone^67^. **b** Schematic diagram of the mechanism of bone toughness^6^. Figures adapted with permission from refs. ^6,67^

The superiority of natural composites to synthetic engineered materials lies in the fact that their overall mechanical properties are invariably far better than those of their individual constituents^4,6^. In this respect, the key feature of natural composites is their hierarchical structure consisting of distinct structural features from the nano- to macrolevel^6^. Synthetic materials often have difficulty achieving both strength and toughness^4,14^. The reason why cortical bone can maintain its strength and toughness is dependent upon its unique multilevel structure^4^. Bone matrix has a multilevel structure from the nano- to macrolevel, which can be divided into 7 levels, 9 levels, and 12 levels according to different classification standards^7,67–69^. The 9-level structure of bone proposed by Reznikov N is shown in Fig. 5a.

Type I Col has a special triple helix structure, which consists of two α1 chains and one α2 chain. Col molecules are ~1.5 nm in diameter and 300 nm in length. The electrostatic attraction of the polar and apolar regions of adjacent Col molecules leads to their polymerization with each other, followed by head-to-tail apposition, parallel alignment, and self-assembly to form the supramolecular structures that constitute the Col fibrils. The parallel Col molecules in a Col fibril are staggered longitudinally by 1/4 of the molecular length (~67 nm). HA crystals (~50 nm × 50 nm × 2 nm) are deposited in these gaps^70,71^ and on the surfaces of Col fibrils^72^ to form mineralized Col fibrils. Several mineralized Col fibrils are combined into bundles by proteoglycans, glycoproteins and other substances and finally polymerized to form mineralized Col fibers. The bone lamellae are composed of mineralized Col fibers and HA. The thicknesses of different types of bone lamellae are different (3–7 μm), and the Col fiber bundles in a single bone lamella have a certain arrangement direction. Cortical bone is mainly composed of osteon and interstitial bone. The osteon is composed of 4–20 layers of annular bone lamellae around the Haversian canal. As the basic structural unit, the bone lamellae are arranged in layers, and the fibers in adjacent bone lamellae are arranged at a certain angle (such as 30°) or are perpendicular to each other. The diameters of the Haverard canal and osteon are ~90 μm and 200 μm, respectively. The Haversian canal is at an angle of 11°–17° with respect to the long axis of the bone. Adjacent Haversian canals are connected by the nearly transverse Volkmann canal. The Harvard canal is also known as the central canal, in which blood vessels are distributed and connected with the blood vessels in the Volkmann canal. The blood vessels in each Haversian canal are connected with each other to form a network. The interstitial bone lamellae are located between the osteon and are composed of several layers of bone lamellae arranged in parallel. They are irregular in shape and have no Haversian tube or blood vessels. They are the residual of the previous osteon. The structure between the osteon and interstitial bone lamella is called the cement line, with a thickness of ~5 μm. Cement lines are not traversed by Col fibers and represent the weakest material in bone, which explains the observation that microcracks tend to follow cement lines rather than crossing over osteons or interstitial lamellae^73^. Cortical bone is distributed on the surface of the respective bone and in the diaphyseal locations of long bones, where it is thicker at the diaphysis, playing a protective and supportive role in the human body.

The special multistage structure of the bone matrix gives it a unique toughening mechanism (as shown in Fig. 5b). Maximilien EL^69^ noted that the integrity of the cortical bone structure arose through a combination of intrinsic toughening mechanisms that acted on the crack tip and extrinsic toughening mechanisms that acted on the crack tail. Internal toughening mechanisms operated at the hierarchical micro- or nanolevels and included the inelastic deformation of the Col mineralized complex, slipping between Col fibrils, sacrificial bonds, etc. Extrinsic toughening mechanisms operated at the micro- and sublevels and mainly included restrictive microcracks, Col fibril bridging, tether bridging and crack deflection/torsion. Intrinsic toughness refers to a material’s inherent ability to resist elastic and plastic deformation, the material basis of which is the structural features of the material’s nanoscale hierarchy, and intrinsic toughness acts to mitigate damage by producing plastic deformation at the crack tip. The extrinsic toughening mechanism mainly occurred at the crack tail, which shielded the driving force of crack propagation and prevented crack propagation. The extrinsic toughening mechanism did not alter the inherent internal toughening properties of the material; it was exerted through the characteristic structure of some hierarchical microlevels on the crack path.

**Fig. 6:**
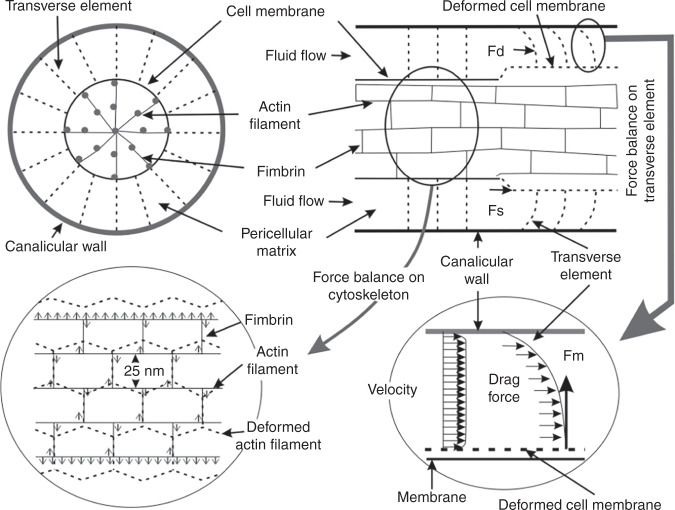
Schematic model showing the structure of the pericellular matrix, the intracellular actin cytoskeleton inside the process and the connection between the pericellular matrix and the intracellular actin cytoskeleton^17^. Figures adapted with permission from ref. ^17^

In addition, other peculiar structures present in the bone matrix that play important roles in the maintenance of bone health also deserve attention, such as in the strain-amplification hypothesis proposed by Cowin SC^74^. Tissue-level strains induced by exercise tend not to exceed 0.2%, while in vitro studies demonstrate that tissue-level strains of 0.2% are unable to initiate intracellular signaling, strains capable of inducing intracellular signaling should be >0.5%, and strains >0.5% may cause damage to bone tissue^75–77^. Cowin SC^74^ considered that whole tissue strains needed to be substantially amplified to elicit a cellular biochemical response. Osteocyte processes were tethered to their canalicular walls by transverse elements that spanned the pericellular space. Fluid flow through the lacunar-canalicular porosity created a tension on the tethering elements that amplified whole-tissue strains by a factor of 10–100 depending on the loading frequency, producing strains large enough to elicit biochemical responses in vitro^17,77,78^. The strain-amplification model is shown in Fig. 6.

## Mechanobiology of the bone matrix

In providing the necessary mechanical support for the human body, the bone matrix will also be affected by many factors in its surrounding environment owing to body movement and other reasons^79–82^. Mechanical stimulation is one of the most important environmental factors. Shuai et al.^15^ summarized the induction mechanisms of osteogenesis by physical stimuli, including electrical, magnetic, and mechanical stimuli. Among them, mechanical stimulation mainly included compressive stress, tensile stress, and FSS. Shuai et al.^15^ explained the bone formation-induced mechanism of mechanical stimulation as follows: (1) When stimulating bone cells, mechanical stimulation can activate various signaling pathways and convert extracellular mechanical signals into corresponding biochemical signals, such as Wnt receptors, integrins, insulin-like growth factor, G proteins and calcium ion channels^77^, thus inducing a series of gene expressions and promoting osteoblast proliferation, differentiation and apoptosis^83,84^. (2) Mechanical stimulation can activate calcium ion channels in the cell membrane, induce the influx of extracellular calcium ions into the cell and increase the intracellular calcium ion concentration, thus promoting bone healing^85,86^. (3) The pressure wave generated by ultrasound can enhance the fluid flow in fracture areas and increase the supply of nutrients and the removal of metabolites, thus promoting the proliferation and differentiation of osteoblasts and fibroblasts^87^. (4) Bone tissue has an abundance of interconnected microchannels, and mechanical stress may generate a strain gradient and cause ion current flow along these microchannels^88^. The strain-generated potentials have been measured in bone samples ex vivo, but their in vivo effects remain to be confirmed by further research.

**Fig. 7:**
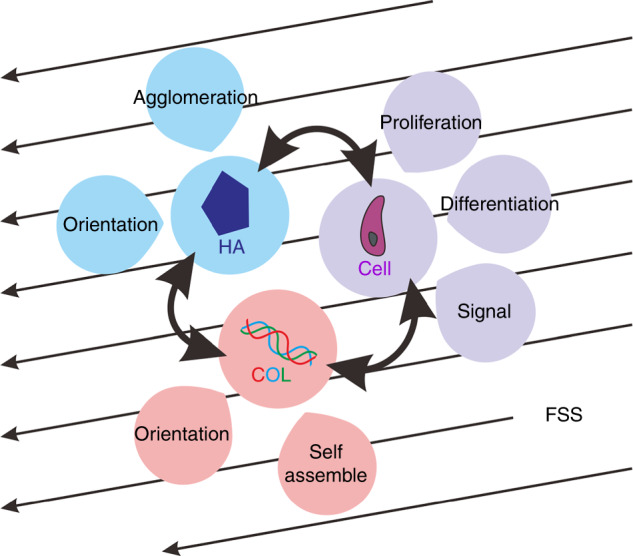
Mechanobiology of the bone matrix. Col, HA and bone-related cells were exposed to FSSs of 0.5 to 3.0 Pa

In the ordered structure of bone, there is a special porous network structure formed by the lacuna in the cancellous bone marrow cavity, bone tube, and bone trabeculae. The porous network structure of bone is filled with tissue fluid, and a mechanical load applied to bone will cause a volume change in these voids and then form hydraulic pressure to promote fluid flow. The fluid flow generates FSS, and the value of FSS in the space between the canalicular wall and the osteocyte process is ~0.5–3.0 Pa. In addition, the Col and HA in newly formed osteoid and those lining the bone surface can also be exposed to fluid flow. Therefore, studying the effects of FSS on Col, HA, and bone-related cells, as well as the interactions between them, can be beneficial for the defect repair and health maintenance of bone tissue^25,30,89^. In this section, the effect of FSS on the bone matrix will be analyzed from four aspects: the mechanobiology of Col, mechanobiology of HA, mechanobiology of mineralization-related cells and the relationships between them (Fig. 7).

### Mechanobiology of Col

**Fig. 8:**
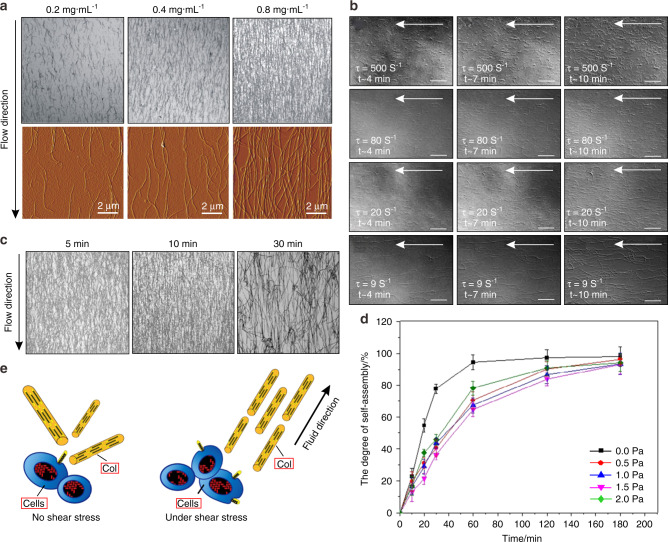
In an in vitro system without cells, the orientation of Col fibers along the flow directions. (**a**)^90^ and FSS can promote the orientation of Col fibrils at a certain size (**b**)^91^ and a certain action time (**c**)^90^. The fibril density increased with increasing concentrations of Col solution. Confocal reflection microscopy (first row) and atomic force microscopy (second row) images of Col fiber matrices prepared at concentrations of 0.2 mg·mL^−1^, 0.4 mg·mL^−1^, and 0.8 mg·mL^−1^ with a flow rate of 11 mL·min^−1^ and conditioning time of 5 min (**a**)^90^. The orientation is optimal at a conditioning time of 10 min (**c**)^90^. Col fibril self-assembly at 500, 80, 20, and 9 S^−1^ shear rates, and the arrangement of Col fibrils is optimal at a shear rate of 20–80 S^−1^ (**b**)^91^. **d** Degree of Col self-assembly over 0, 0.5, 1.0, 1.5, and 2.0 Pa FSS for 10, 20, 30, 60, 120, and 180 min, respectively^11^. **e** Effects of FSS on Col. FSS can promote the orientation of Col fibers with an increasing shear rate within a certain range. In addition to the Col fiber arrangement, FSS can also induce Col fibril expression, making the Col fiber diameter distribution more uniform. Figures adapted with permission from refs. ^11,90,91^

Obviously, in in vitro systems, FSS can influence Col fibril orientation (Fig. 8a) and self-assembly (Fig. 8d). The influences of FSS on the arrangement of Col fibrils showed that the orientation degree of Col fibrils increased with increasing shear rate, and the Col fibril density increased with an increasing concentration of Col solution (Fig. 8a)^90^. Moreover, a 10 min action time of FSS was better for the alignment of Col fibers (Fig. 8c)^90^. Certainly, the effect of FSS on Col self-assembly does not follow a trend of the larger the better. The study of the self-assembly of type I tendon Col under FSS showed that the axial growth rate of Col fibers was the highest at the lowest shear rate. In contrast, the greater the shear rate is, the slower the fibril growth, and the arrangement of Col fibrils is optimal at a shear rate of 20–80 S^−1^ (Fig. 8b)^91^. According to the effects of FSS on Col fibril orientation, a three-dimensional arrangement of Col fibers could be prepared by microfluidic techniques^92^. In one such study, five three-layer models were used to induce different orientation levels of Col fibrils. The orientation of Col fibrils was shown to increase in response to shear stress, and the mechanical properties of Col were also different according to its different orientation degrees^58^. By further studying the effects of shear stress on the microstructure of Col fibers, it was found that the Col bearing capacity was dependent upon the conversion of the shear stress between fibrils or other-oriented tissues^93^. However, it is not clear whether shear forces were converted into other forms of force or attenuated by changing the structure. The direction of Col fibrils arranged by FSS in vitro is not stable and cannot achieve a highly ordered arrangement, which is consistent with natural Col fibrils. Therefore, it is necessary to study the structure of Col after FSS loading and combine the shear stress with the surface energy distribution of Col fibrils to study mineralized Col, the structure of which is close to a naturally highly ordered arrangement.

Osteoblasts are able to secrete Col during the remodeling of bone, and this process can be regulated by FSS. That is, in addition to Col fiber arrangement, FSS can also induce Col fibril expression (Fig. 8e)^94^^,^^95^. In one such study utilizing FSS that occurred for 0.5, 1.0, 2.0, or 4.0 h, shear stresses of 1.2 Pa were used, and the transcription and secretion of type I Col and the activity of alkaline phosphatase were shown to increase in response to shear stress. Under larger stresses, the diameter of Col fibrils is more balanced, and the density of fibrils is lower. At lower stresses, the size distribution of Col fibrils is wider^96^. Overall, Col fibrils have good mechanobiology, which, along with its specific orientation and mechanical properties, can be obtained by controlling the size, direction and loading mode of FSS.

### Mechanobiology of HA

Similarly, HA is an important inorganic matrix in bone and is associated with the formation and growth of crystals during the biomineralization process of bone. As a typical form of stress in bone, FSS can affect the entire crystallization process of HA.

A biomineralization study found that in the formation process of HA crystals, the first step was the formation of amorphous calcium phosphate (ACP) precursors, and then the ACP precursor transformed into HA crystals accompanied by sustained calcium and orthophosphate ion release^97–99^. Therefore, the effects of FSS on the formation and growth process of ACP and HA are of great significance for biomineralization. Lee et al.^100^ and Park et al.^101^ showed that shear stress can induce the crystallization of amorphous materials, improve crystallinity, and improve the mechanical properties of nanocrystalline materials. Furthermore, different sizes and load times of shear stress application are critical factors for crystallization^102^. The influence of shear stress on crystallization is also studied by means of molecular dynamics simulations and subsequent cluster analyses. Ling et al. showed that the high-molecular-weight components in the blends could act as a template for crystallization under shear stress, and FSS could induce low-molecular-weight polyethylenes to form different crystal precursor structures^103^.

**Fig. 9:**
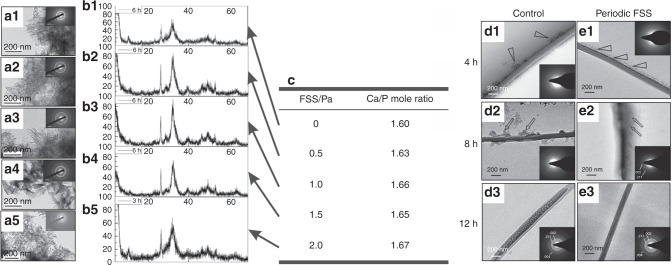
Effect of FSS on conversion of ACP to HA. **a**–**c** In vitro system without cells, morphologies and crystal characteristics of ACP converting into HA after aging for 6 h^12^. Transmission electron microscopy (TEM) and X-ray diffraction characterization of HA under 0, 0.5, 1.0, 1.5 and 2.0 Pa FSS environments, respectively, with select area electron diffraction patterns in the top right corner of each TEM image. **d**, **e** In an in vitro system without cells, periodic FSS can inhibit the emergence of large clusters and promote the intrafiber mineralization of apatite^13^. Figures adapted with permission from refs. ^12,13^

In the ACP crystallization process, the effects of shear stress on the conversion of ACP into HA crystals and the structure of HA crystals were further investigated. Since the expansion of the crystal region requires the consumption of peripheral calcium ions and orthophosphate ions and releases hydrated protons, the mechanical strength of the inner region is decreased. Under the action of FSS, the initial particle digestion is accelerated, and the microcrystals produced by the crystallization at multiple sites cause the calcium phosphate to precipitate quickly^104^. Recently, our group found that low shear stresses (≤1.0 Pa) favored the transformation of ACP crystals and accelerated the formation of ordered calcium-deficient HA structures. The resulting crystal structure was longer and straight and had an orientation trend. However, high shear stresses (>1.0 Pa) were ineffective, and the resulting calcium-deficient HA structure was impaired (Fig. 9a–c). Moreover, bioactivity evaluation showed that the longer, straight and aligned crystals had better cell compatibility and positive effects on the mechanical properties and biological properties^12^. In addition, FSS can inhibit the emergence of large clusters and promote the intrafiber mineralization of HA (Fig. 9d, e)^13^. In summary, the results revealed two effects of shear stress on the crystallization process. On the one hand, shear stress could promote the formation of separated crystallites and suppress the appearance of large clusters. On the other hand, shear stress could induce microcrystalline growth and orientation along a certain direction^105^. However, HA crystals are mainly presented in the form of lamellar structures in native bone. Under the action of FSS, how to obtain lamellar HA crystals deserves in-depth studies.

### Mechanobiology of mineralization-related cells

Bone tissue comprises several cell types, including precursor cells, osteoclasts, osteoblasts, osteocytes and bone-lining cells. In addition, the modeling and remodeling processes of bone are also accompanied by angiogenesis and neurogenesis^106^. All the mineralization-related cells involved in the modeling and remodeling of bone are able to significantly respond to FSS^107,108^. The effects of FSS on mineralization-related cells are mainly reflected in cell proliferation, differentiation, signal expression and signaling pathways^109,110^. Due to the variety of cell secretions and the complexity of signal pathways, many studies have proved the complex mechanobiology of mineralization-related cells under FSS^111–114^. Correspondingly, different sizes, times and loading methods of FSS will have different effects on cell behavior^115–117^.

Because of the substantial role of FSS, numerous studies have focused on the influences of FSS on increasing the proliferation and differentiation of mineralization-related cells^111^. FSS promoted the differentiation of human mesenchymal stem cells into osteoblasts^112–115^. Li et al.^116^ and Sikavitsas et al.^118^ cultured osteoblasts using a three-dimensional perfusion bioreactor and found that the mineralized matrix deposition of bone marrow stromal osteoblasts increased with increasing FSS. Further studies have shown that FSS can coincrease the differentiation of osteoblasts with the extracellular matrix^119^, rearrange the orientation of osteoblasts and promote the expression of growth factors as well as ultimately enhance the differentiation ability of bone^120^. Furthermore, the effects of shear loading methods on the proliferation of cells were investigated. Multidirectional FSS could inhibit osteoclast activity, maintain osteoblast function and increase osteoblast alkaline phosphatase activity^121^. Periodic FSS is more effective in promoting osteoblast proliferation and the phosphorylation of extracellular signal-regulated kinase-5 (ERK5) in cells than continuous FSS^122^.

**Fig. 10:**
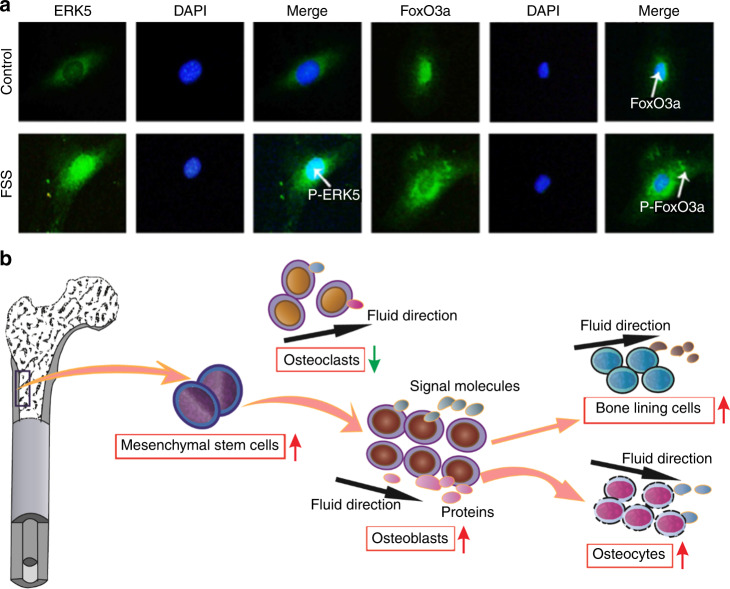
Effects of FSS on cell differentiation and gene expression. **a** FSS can affect the signal expression in MC3T3-E1 cells. The nuclear translocation of P-ERK5 and FoxO3a; FoxO3a nuclear translocation (green fluorescence) and nuclear location (blue fluorescence)^132^. **b** Mechanism of FSS on mineralization-related cells. FSS can promote mesenchymal stem cell differentiation into osteoblasts and then promote the further differentiation and orientation of osteoblasts, bone lining cells and osteocytes. More, it can induce the expression of signaling molecules and proteins. Figures adapted with permission from ref. ^132^

Because the process of Col mineralization is regulated by many growth factors, significant changes in signal expression and signaling pathways have also been observed after exposure to shear stress^123–126^. In previous studies, when FSS was applied to osteoblasts, it induced higher signal expressions of prostaglandin E2, transforming growth factor-β, inositol trisphosphate, cyclic adenosine monophosphate (cAMP), nitric oxide (NO) and so on within a certain range^112,127–129^. A study utilized a flow that occurred for 5, 30, or 120 min every other day for 20 days, shear stresses of 0.16 Pa were used, and osteopontin and bone sialoprotein expression were shown to increase in response to shear stress^113^. The cyclooxygenase-2 (COX-2) mRNA expression levels were shown to improve when osteoblastic differentiation was observed at a higher and longer action time of FSS; in contrast, the receptor activator of nuclear factor kappa B ligand (RANKL)/osteoprotegerin (OPG) mRNA expression levels were shown to decrease^114^. One of these studies also showed that the effects of cyclic FSS on OPG and RANKL protein expression in osteoblasts were better than those of continuous FSS^130^. Signaling pathways and signaling molecules are closely related to mineralization^131^. In addition, FSS can also be involved in the inhibition of the tumor necrosis factor-α-induced apoptosis of cells through the regulation of the ERK5-AKT-forkhead Box O 3a (FoxO3a)-Bim/Fas ligand signaling pathway (Fig. 10a)^132^. With the thorough study of signaling pathways, scientists have studied the effects of different loading methods on cell differentiation, proliferation, metabolism signal transduction and signaling expression. Cyclic strain could enhance the matrix mineralization of adult human mesenchymal stem cells via the extracellular signal-regulated kinase signaling pathway and upregulate bone morphogenetic protein-2 expression through mitogen-activated protein (MAP) kinase and COX-2/PGE2 signaling pathways in human periodontal ligament cells^133,134^. Specifically, some researchers found that fluid shear-induced intracellular calcium transients played an important role in the mechanical transmission of osteoblasts. First, FSS induced the depolarization of the membrane and then transiently increased intracellular calcium ([Ca^2+^]_*i*_) in osteoblasts by activating tension-sensitive ion channels and voltage-sensitive Ca^2+^ channels, ultimately promoting cell calcification^135^. In addition to the expression of signaling molecules and signaling pathways, FSS can also promote the uptake of polystyrene nanoparticles into cells, and the uptake amount reached a maximum when the shear stress was 0.05 Pa and then decreased with increasing shear stress^136,137^. Similarly, mesenchymal stem cells were able to infiltrate into an electrospun poly(lactic-co-glycolic acid) scaffold better under FSS^138^.

In addition, cell junctions also play important roles in cell communication, which can be affected by FSS. Gap junctions are found in all kinds of bone-related cells, especially in osteoblasts and osteoclasts^139^. Osteoblasts and osteoclasts express a variety of connexins, including Cx40, Cx43, Cx45, Cx46, and Cx37^139^. Cx43 is a highly expressed gap junction protein in bone. The expression of Cx43 protein in MLO-Y4 cells is regulated by fluid stress stimulus^140–142^. Alford AI’s study showed that the phosphoserine content of Cx43 in MLO-Y4 cells exposed to oscillating flow for 1 h increased approximately twice as much as that in MLO-Y4 cells without flow treatment^142^. Primary cilia have been demonstrated to participate in osteocyte mechanotransduction both in vitro and in vivo^143^. The research of Malone AMD et al. showed that primary cilia influenced osteocyte cellular responses to external shear stress by regulating the intracellular cAMP levels and extracellular calcium entry independent of intracellular calcium signals^144,145^.

In summary, FSS can directly or indirectly affect the expression, function and distribution of connexins on cell surfaces and also affect the synthesis, metabolism and release changes of growth factors, thus affecting the biological behavior of effector cells. Mineralization-related cell behavior influenced by an FSS environment is shown in Fig. 10b. However, how chemical signals affect mechanical sensitivity is unclear, and the mechanism by which high levels of FSS inhibit cells has not yet been resolved.

### Effects of mechanical stimulation on the interactions between the bone matrix and cells

**Fig. 11:**
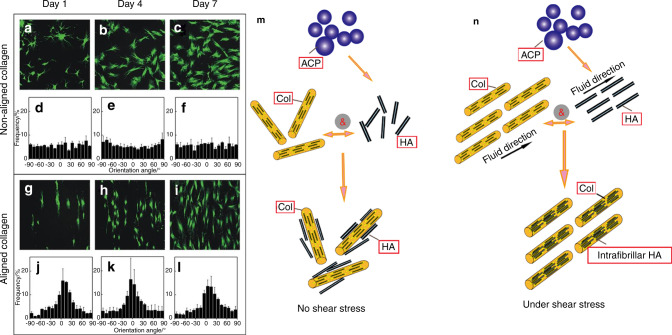
Effect of mechanical stimulation on the interaction between bone-related cells, Col and HA. **a**–**l** Influence of aligned and randomly aligned ECM structures on cell morphology and orientation^154^. Viable mesenchymal stem cells (green) adopting random and oriented morphologies on nonaligned (**a**–**c**) and aligned Col (**g**–**l**) on Days 1, 4, and 7. Quantification of cell orientation angles with the corresponding images is shown in the panels below (**d**–**f**, **j**–**l**). (Scale bar: 200 μm; ±s.d., *n* = 4 replicates from two different donors). **m**, **n** Effects of FSS on Col/HA mineralization. Col is a template for mineralization, and HA crystals grow along the long axis of Col fibrils. FSS can promote ACP precursor transformation into HA crystals and improve the crystallization and orientation of HA. Figures adapted with permission from ref. ^154^

Bone, as a compound formed by the ordered arrangement of Col and HA, in addition to the effects of FSS on Col and HA, respectively, is also affected by the interactions between cells, Col fibrils and HA crystals^146,147^. Many researchers have suggested that cells are the driving force behind matrix anisotropy and can modulate the synthesis and degradation of Col fibers under mechanical stress^148–150^. Furthermore, there is another mechanism, that of the orientation of Col fibrils, which can be regulated by mechanical stress and subsequently induce the orientation, proliferation and signal transmission of cells^151^. The oriented mineralized Col fibrils also play an important role in tissue mineralization. Our previous study demonstrated that mechanical strain combined with an HA/Col composite could obviously induce the differentiation of mesenchymal stem cells into osteoblasts, which had a better effect than mechanical strain or HA/Col composite treatment alone^152,153^. Researchers used the simple technique of spin coating to produce highly aligned arrays of Col fibers, and then by a simple modification method produced orthogonal Col lamellae, which are very common in loaded-bearing tissues. The results of cultured corneal fibroblasts with regard to these aligned Col fibers showed that cells grew along the orientation of Col fibrils (Fig. 11a–l)^154^. Similarly, the neatly arranged Col matrix contributed to the differentiation of both mesenchymal stem cells and precursor cells in multiple directions and then guided the process of matrix mineralization and the morphogenesis of anisotropic tissues^92^. Furthermore, Kemeny et al. showed that when culturing cells on aligned Col fibers, a variety of cell behaviors were carried out along the orientated Col fibrils, such as the migration, growth and differentiation of cells as well as the expression of signal molecules^155^.

Moreover, the nucleation and growth of HA crystals preferentially oriented parallel to the orientation direction of the oriented Col (Fig. 11m, n)^156,157^. Owing to the calcium-phosphorus along the charged side of Col fibril chains that nucleated, crystallized and orientated, the oriented Col fibrils could help the HA obtain a highly oriented and homogeneous crystal structure^158,159^. The HA crystal c-axis aligned with the long axis of the Col fibrils, which is the same as the Col fibril and HA crystal arrangement in native-like bone^160^. The mineralization of HA on amphiphilic polypeptide self-assembled nanofibers also induced crystal growth along the long axis of the fibrils^63^. The self-assembly of Col fibrils arranged in a more ordered manner could change calcium phosphate mineralization, nucleation and growth, which is a major step for in vitro biomimetic preparation^64^. Furthermore, in the orientation of Col fibrils in different directions, the interaction between Col and HA was significantly different for the orientation dependence of Col mechanics^65^. The conversion of ACP, the secretion of Col fibrils and their orientation are better in an FSS environment. Correspondingly, regarding how HA affects the structure and properties of Col, some studies have shown that HA can improve the mineralized Col complex toughness and influence the deformation behavior of Col by affecting the connection between Col molecules and water^57,58,161,162^. In addition, some studies found that a nanointerface arrangement could enhance the mechanical strength of Col/HA mineralized materials^163^. By stretching Col in the vicinity of different crystal surfaces of HA, the orientation dependence of Col was different^164^.

## Summary and outlook

This paper summarized the biomechanical properties of the bone matrix, discussed the biological significance of the mechanical properties of the bone matrix, analyzed the components and structural basis of the bone matrix with regard to these mechanical properties, and studied the effects of mechanical stimulation, especially FSS, on the components of the bone matrix, cells and their interactions. The bone matrix exhibits both high strength and strong toughness. There are obvious mechanical differences between cortical bone and cancellous bone, but the mechanical differences between cortical bone or cancellous bone in various parts of the human body are not obvious. Cortical bone has obvious mechanical anisotropy, while cancellous bone does not. The mechanical properties of the bone matrix are related to its function in the human body. For example, the mechanical properties of cortical bone, which undertakes the main mechanical support function, are significantly stronger than those of cancellous bone. The biomechanical properties of the bone matrix change with the diameter distribution, crosslinking degree, orientation, denaturation and aging degree of Col fibers. The crystal size, microstrain and calcium–phosphorus ratio of HA have obvious effects on the density and mechanical strength of bone. The mineralization degree of Col and the mineralization position of HA can also affect the biomechanical properties of the bone matrix. The effect of FSS on Col is mainly manifested in the directional arrangement and self-assembly of Col fibers. The effect of FSS on HA is mainly manifested in promoting the formation of separated crystallites, suppressing the appearance of large clusters as well as inducing microcrystalline growth and orientation in a certain direction. The effects of FSS on bone-related cells are mainly characterized by cell proliferation, differentiation, signaling pathways and signal expression. The effect of FSS on the interactions between these components is mainly reflected in the fact that while FSS promotes the directional arrangement of Col fibers, it leads to the directional crystal growth of HA along the same direction of Col fiber orientation. Moreover, a series of cell growth behaviors on directional mineralized Col fibers also proceed along the direction of Col fiber orientation, such as cell migration, growth and differentiation and the expression of signaling molecules.

Therefore, the following points deserve attention in future research:

1. Most of the traditional methods for testing the mechanical properties of the bone matrix are destructive, which will cause damage to human bones and are not suitable for clinical use. It is necessary to improve the accuracy of the existing nondestructive testing methods and develop new nondestructive bone testing methods.
2. The effects of the micro-/nanostructure of HA on the mechanical properties of the bone matrix need to be further studied, such as the ratio of HA to Col, the crystallization position of HA on Col fibers (in or out of fibers), and the crystallization degree and crystal structure of apatite.
3. Diseases often cause a series of changes in the composition and structure of the bone matrix, which in turn lead to changes in the mechanical properties of the bone matrix. How to detect, diagnose and treat bone tissue diseases by observing the changes in the composition and structure of the bone matrix and the overall mechanical properties of the bone matrix needs to be further studied.
4. Previous studies have given more attention to the effects of the mechanical environment on bone-related cells. The mechanisms of the effects of the mechanical environment on the bone matrix have not received due attention, and equal attention should be given to the regulation of the bone matrix by the mechanical environment in future studies.
5. More attention should be given to the dynamic regulation of the mechanical environment on bone-related cells and bone matrix; that is, mechanical stimulation can regulate not only the secretion of the extracellular matrix by affecting the function of bone-related cells but also the biological functions of cells by directly changing the microstructure and performance of the bone matrix.
6. When investigating the mechanobiological response of the bone matrix, consideration should be given to how to construct bioactive bone repair materials using bioreactors in vitro in the fields of bone tissue engineering and regenerative medicine.
